# Phase diagram and spin mixing dynamics in spinor condensates with a microwave dressing field

**DOI:** 10.1038/srep14464

**Published:** 2015-09-25

**Authors:** Yixiao Huang, Wei Zhong, Zhe Sun, Zheng-Da Hu

**Affiliations:** 1 School of Science, Zhejiang University of Science and Technology, Hangzhou, Zhejiang, 310023, China; 2 Laboratory of Quantum Engineering and Quantum Materials, SPTE, South China Normal University, Guangzhou 510006, China; 3 Department of Physics, Hangzhou Normal University, Hangzhou 310036, China; 4 School of Science, Jiangnan University, Wuxi 214122, China

## Abstract

Spinor condensates immersed in a microwave dressing field, which access both negative and positive values of the net quadratic Zeeman effect, have been realized in a recent experiment. In this report, we study the ground state properties of a spinor condensate with a microwave dressing field which enables us to access both negative and positive values of quadratic Zeeman energy. The ground state exhibits three different phases by varying the magnetization and the net quadratic Zeeman energy for both cases of ferromagnetic and antiferromagnetic interactions. We investigate the atomic-number fluctuations of the ground state and show that the hyperfine state displays super-Poissonian and sub-Poissonian distributions in the different phases. We also discuss the dynamical properties and show that the separatrix has a remarkable relation to the magnetization.

After successful experimental realizations of spinor condensates in ^23^Na and ^87^Rb atoms^1^,^2^,^3^, experimental and theoretical studies on spinor condensates have emerged as one of the most fast moving frontiers in degenerate quantum gases. An optical trap enables simultaneous and equal confinement of atoms in different hyperfine states. In comparison to scalar condensates, spinor condensates can exhibit richer quantum phenomena due to their internal spin degrees of freedom. In addition to Feshbach resonances and optical latices which tune the interatomic interactions, spinor condensates systems are offering an unprecedented degree of control over many other parameters, such as the spin, the temperature and the dimensionality of the system^4^,^5^.

During the past few years, many researches have demonstrated the mean field (MF) ground state and the dynamics of spinor condensates by holding the Bose-Einstein condensate with a fixed magnetic field^6^,^7^,^8^,^9^. Coherent spin mixing dynamics has also been observed in terms of the population oscillation in different Zeeman states inside spinor condensates, such as *F* = 1 hyperfine spin states of ^23^Na condensates^6^,^7^,^8^,^9^,^10^,^11^,^12^ and both *F* = 1 and *F* = 2 hyperfine spin manifolds of ^87^Rb condensates^13^,^14^,^15^,^16^,^17^. Due to the interconversion among multiple spin states and magnetic field interactions, many interesting phenomena have been theoretically and experimentally demonstrated in spinor condensates, such as quantum phase transition^18^,^19^,^20^, quantum number fluctuation^21^, spin population dynamics^22^,^23^,^24^,^25^,^26^,^27^,^28^,^29^,^30^,^31^,^32^ and spin nematic squeezing^33^. However, for spin-1 condensate, the magnetic field can only introduce a positive net quadratic Zeeman energy where *δ*_net_ ∝ *B*^2^ > 0. Recently, many methods have been explored for degenerating both positive and negative quadratic Zeeman shifts, such as through a microwave dressing field^4^,^11^,^34^,^35^,^36^,^37^,^38^ or via a linearly polarized off-resonant laser beam^39^. With the microwave dressing field, the value of quadratic Zeeman shift can be swept from −∞ to +∞ in the present experiment^38^. Meanwhile, the quantum phase transition in the spinor condensates with antiferromagnetic interactions have been investigated by adiabatically tuning the microwave field^40^.

In this report, we study the ground state properties of a spin-1 condensate with a microwave dressing field where both negative and positive values of *δ*_net_ can be accessed. The phase diagrams of the ground state for both ferromagnetic ^87^Rb and antiferromagnetic ^23^Na interactions are demonstrated. Based on the fractional population *ρ*_0_ of the hyperfine state *m*_*F*_ = 0, we define three distinct phases with *ρ*_0_ = 0, 0 < *ρ*_0_ < 1, and *ρ*_0_ = 1 representing the antiferromagnetic (AFM) phase, the broken axis symmetry phase (BA) and the longitudinal polar phase, respectively. By tuning the parameters of *δ*_net_ and the magnetization *m*, quantum phase transitions will occur. We also investigate the atom number fluctuations of the ground state with different values of *δ*_net_ and *m*. It is found that the hyperfine state of *m*_*F*_ = 0 exhibits super-Poissonian distributions in the AFM phase for both ferromagnetic and antiferromagnetic interactions, while sub-Poissonian distributions are presented in the BA phase. At the boundary of BA and AFM phases, the atom number fluctuation attains its maximum value. In the polar phase, the Mandel *Q* parameter equals to −1, which means no atom number fluctuation. For the dynamical properties, we find a remarkably different relationship between the total magnetization |*m*| and a separatrix in the phase space. Finally, we show that our results agree with the prediction that spin dynamics in spin-1 condensates substantially depend on the sign of the ratio between the net quadratic Zeeman effect and the spin-dependent interaction^31^.

## Results

### Model and its requantization

In the second quantized form, the system of an interaction atomic spin-1 Bose gas in the presence of an external field is given by^41^,^42^


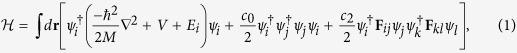


where *ψ*_*i*_ are the field operators for the spin components *i* = 1, 0, −1, *M* is the atomic mass and *E*_*i*_ are the Zeeman energies of the hyperfine states given by the Brei-Rabi formula and *V* the potential. In addition, **F** is the spin-1 matrix. 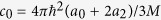 and 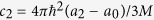 are the collisional interaction parameters for spin independent and spin exchange interactions, respectively. Here *a*_*F*_ are the *s*-wave scattering lengths for two *f* = 1 atoms of total spin angular momentum *F* = 0, 2 in the channel.

With the magnetic field *B*, the linear and quadratic Zeeman shifts are parameterized by 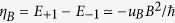 and 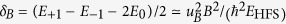, respectively. Here *E*_HFS_ is the hyperfine splitting and *B* is the magnetic field. Since the total atom number and the total magnetization *m* are conserved in the dynamical process, the linear term does not affect the system dynamics. By using a microwave dressing field, the net quadratic Zeeman shifts *δ*_net_ can be expressed as *δ*_net_ = *δ*_*B*_ + *δ*_*M*_ with *δ*_*M*_ induced by the microwave dressing field^12^,^40^. Microwave dressing field is an off-resonant microwave field. In the recent experiment, the microwave dressing field was realized by plus a polarized microwave field with *π*- or *σ*^±^-polarizations in a sodium condensate (^23^Na or ^87^Rb atoms) with a constant and homogeneous magnetic field *B*. Far from any hyperfine resonance, a level shift 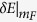 for the state 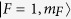 can be found^37^,^38^


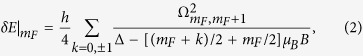


where *k* = 0 or ±1 correspond to the *π*- or *σ*^±^-polarized microwave pulses respectively, and Δ is the detuning of the microwave pulses from the transition between the states 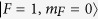 and 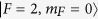. The quadratic Zeeman shift induced by a microwave dressing field then reads as 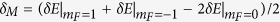, which can be tuned from −∞ to +∞^37^,^38^. For a given *k*, the allowed Rabi flopping is between the states 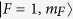 and 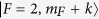 and its on-resonance Rabi frequency 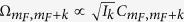 for the *π*- or *σ*^±^-polarized transition, where *I*_*k*_ is the intensity of the *k*-polarized microwave component and 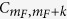 is the Clebsch-Gordan coefficient of the transition.

For both ^87^Rb and ^23^Na atoms, the spin-dependent interaction is much weaker than the density-dependent interaction. As a consequence, one can adopt the single mode approximation (SMA)^23^,^43^,^44^. The effective energy of system can be written as


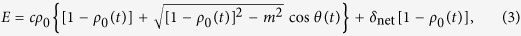


where *ρ*_0_ is the fractional population of the hyperfine state *m*_*F*_ = 0, *m* = *ρ*_1_ − *ρ*_−1_ is the magnetization, and *c* is the renormalized spin-dependent interaction. The cases *c* > 0 and *c* < 0 correspond to antiferromagnetic (^23^Na) and ferromagnetic (^87^Rb) spinor condensates, respectively. *θ* = *θ*_1_ + *θ*_−1_ − 2*θ*_0_ is the relative phase among the three different hyperfine states. The corresponding time evolutions of *ρ*_0_ and *θ* are governed by the following differential equation^22^


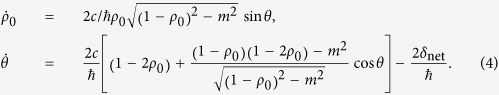


Since *ρ*_0_ and *θ* can be interpreted as the effective conjugate variables, the above equations can also be derived from the canonical equation of Hamiltonian dynamics 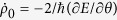 and 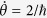

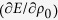
.

### Ground state properties

**Figure 1:**
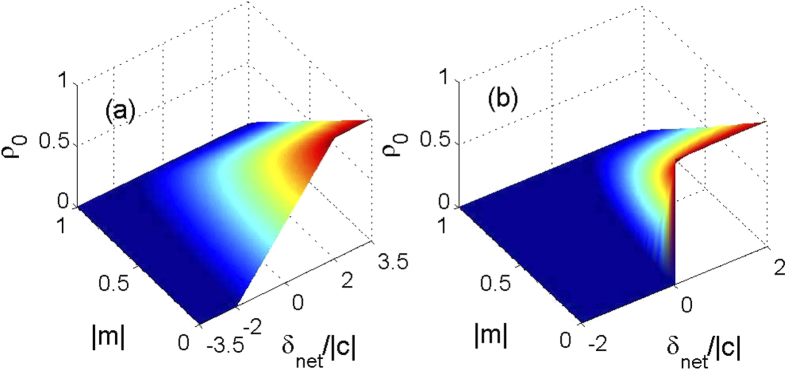
The fractional population in the MF ground state. The fractional population *ρ*_0_ for the ground state in the hyperfine state *m*_*F*_ = 0 versus *δ*_net_ and magnetization *m* for (**a**) ferromagnetic spinor (^87^Rb with *c* < 0) and (**b**) antiferromagnetic spinor condensates (^23^Na with *c* > 0).

**Figure 2:**
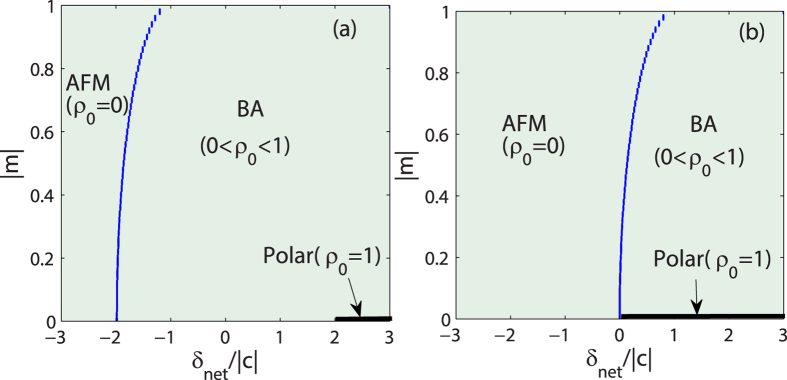
Phase diagram. MF phase diagrams of the spin-1 condensate with (**a**) ferromagnetic and (**b**) antiferromagnetic interactions.

By minimizing the energy, *ρ*_0_ in the MF ground state of the system can be obtained. If 
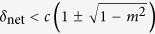
, *ρ*_0_ = 0, and if *m* = 0 and *δ*_net_ > −*c*(1 ± 1), *ρ*_0_ = 1. For all other *m* and *δ*_net_, *ρ*_0_ is the root of the following equation


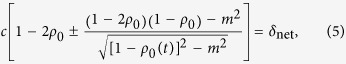


where the sign ± correspond to the cases of ferromagnetic and antiferromagnetic interactions, respectively. As shown in Fig. 1, the fractional population *ρ*_0_ in the MF ground state as a function of |*m*| and *δ*_net_ is plotted for both antiferromagnetic (^23^Na) and ferromagnetic (^87^Rb) spinor condensates. It can be seen from Fig. 1(a) that, for the case of ferromagnetic interactions, the value of *ρ*_0_ may start to become nonzero at *δ*_net_ = −2|*c*| and grow to its maximum as *δ*_net_ is increased. Particularly, *ρ*_0_ = 1 at *δ*_net_ ≥ 2|*c*| when *m* = 0. For the case of antiferromagnetic interactions shown in Fig. 1(b), a sharp jump for the value of *ρ*_0_ near *δ*_net_ = 0 takes place. When *δ*_net_ < 0, *ρ*_0_ = 0 for all values of |*m*|. When *δ*_net_ > 0, there exists a region of values of |*m*| in which *ρ*_0_ > 0 and especially *ρ*_0_ = 1 for *m* = 0. According to the behavior of *ρ*_0_ in the MF ground state, the phase diagrams are plotted in Fig. 2 for both cases of ferromagnetic and antiferromagnetic interactions. There are three different phases in the MF ground state, i.e., the longitudinal polar phase (*ρ*_0_ = 1), the AMF phase (*ρ*_0_ = 0), and the BA phase (0 < *ρ*_0_ < 1). When *m* = 0, the order parameter of the AMF phase is 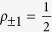, *ρ*_0_ = 0 and that of the polar state is *ρ*_0_ = 1. In the Spherical-harmonic representation, the former state is obtained by rotating the latter about the *x* axis by *π*/2, and therefore, these two states are equivalent and degenerate^4^,^5^. In the BA phase, the ground state features spontaneous breaking of axisymmetry or spontaneous breaking of the SO(2) symmetry^45^. In the recent experiment, the phase diagram for the antiferromagnetic interaction (Fig. 1(a)) has been realized via adiabatically tuning the microwave field^40^.

To study the ground state atom number fluctuations, the effective Hamiltonian will be requantized by treating *ρ*_0_ and *θ* as operators which satisfy the following commutation relation^21^,^46^


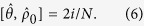


As a result, 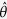 can be represented as 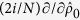 in the 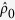 representation. As long as *ρ*_0_ stays away from the two ends at *ρ*_0_ = 0 and 1, 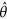 is a Hermitian operator. By symmetrizing the nonlinear term containing both 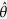 and 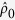 with Wigner-representation technique in conventional phase space, we obtain an effective Hamiltonian^21^,^27^


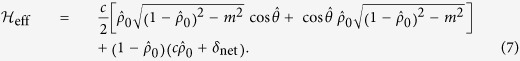


To gain insight to the properties of the ground state, we study the atom number fluctuations in terms of the Mandel *Q* parameter


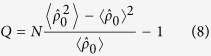


with *Q* < 0, *Q* = 0, and *Q* > 0 specifying sub-Poissonian, Poissonian, and super-Poissonian distributions, respectively. In particular, when *Q* = −1, it means no atom number fluctuation. For a linear harmonic oscillator, the coherent state 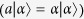 exhibits a Poissonian fluctuation when the number of excitation *a*^†^*a* is counted. The fluctuation level for such a state is also called shot noise.

**Figure 3:**
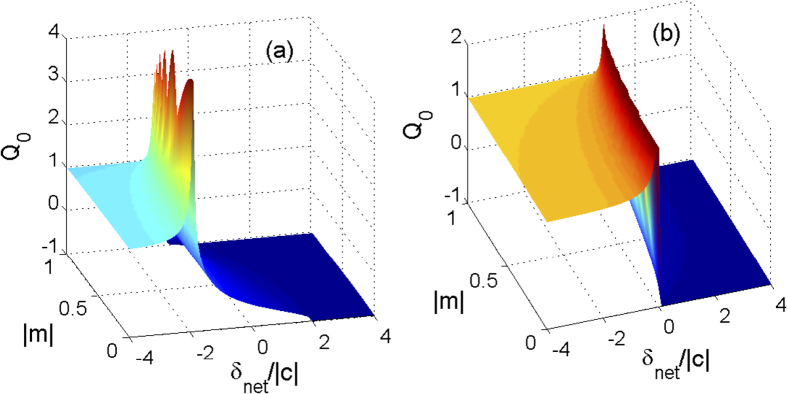
Mandel Q parameters. The Mandel *Q* parameter versus magnetization |*m*| and *δ*_*net*_/*c* for (**a**) ferromagnetic and (**b**) antiferromagnetic interactions with *N* = 400 and |*c*|/*h* = 52 Hz.

In Fig. 3, the Mandel Q parameters are plotted as functions of |*m*| and *δ*_net_ with ferromagnetic and antiferromagnetic interactions. For the ferromagnetic case, in the AFM phase, we find *Q* ≃ 1, which means the hyperfine state *m*_*F*_ = 0 displays a super-Poissonian distribution. At the boundary between the AFM and BA phases, *Q* attains its maximum. In the BA phase, we can see *Q* decreases with increasing the parameter *δ*_net_ for a fixed magnetization |*m*|. In this phase, we can see *Q* < 0 except near the boundary between the two different phases, which indicates the ground state exhibits a sub-Poissonian distribution. In the BA phase, we can also find the value of *Q* parameter shows a sudden transition from *Q* > −1 to *Q* = −1. It is due to the fact that when the quadratic Zeeman shift *δ*_net_ is large enough, *ρ*_0_ keeps constant as *ρ*_0_ = 1 − |*m*|. In such a situation, there is no atomic-number fluctuations and *Q* = −1 indicating the polar phase. For the antiferromagnetic interactions, the behavior of *Q* is similar to that in the ferromagnetic interactions.

### Dynamical properties

**Figure 4:**
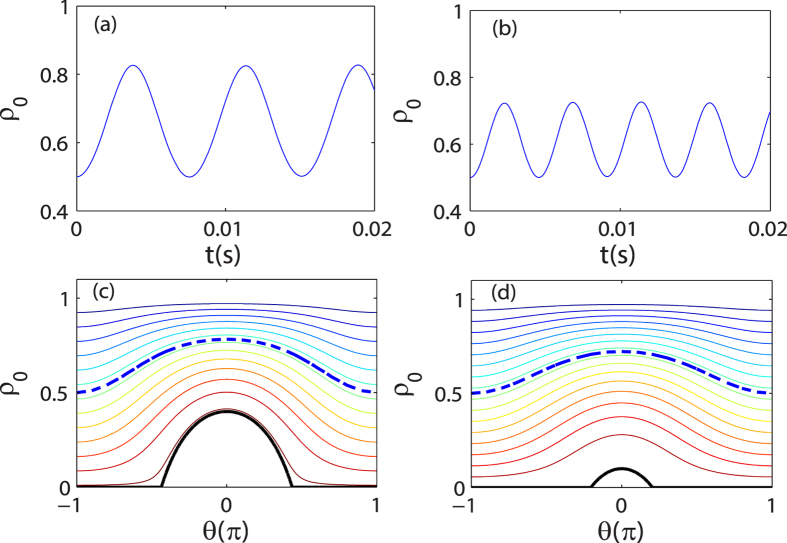
Time evolution of *ρ*_0_ and equal-energy contour. Time evolution of *ρ*_0_ at (**a**) *δ*_net_ = 1.2*c* and (**b**) *δ*_net_ = 1.8*c* with initial condition *m* = 0 and *θ* = 0. The corresponding equal-energy contour plots generated from Eq. (3) for (**c**) *δ*_net_ = 1.2*c* and (**d**) *δ*_net_ = 1.8*c*. The heavy dashed (blue) lines correspond to the energy of the initial state. Heavy black lines represent the energy of the separatrix between oscillating and running phases solutions. The energies of lines decrease from top to bottom. The parameter used here is *c*/*h* = 52 Hz.

Because of the spin-exchange interaction, spinor condensates present dynamical responses significantly which are different from that in scalar condensates. In addition to density excitations or waves, the coherent spin mixing between different spin components will arise in spinor condensates. As shown in Fig. 4(a,b), the time evolution of *ρ*_0_ is plotted as a function of time *t* with different *δ*_*net*_ for antiferromagnetic interactions. We can see *ρ*_0_ exhibits a periodical oscillation. The equal energy contours of Eq. (3) are also plotted in Fig. 4(c,d) for *m* = 0 with the initial condition *θ* = 0 and *ρ*_0_ = 0.5. At any *δ*_net_, we can define a separatrix, i.e., the energy contour *E*_sep_, a point on which is called a saddle point and satisfies the equation 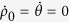. This defines the boundary between the two different regions in phase space. When the initial energy of the system *E*_0_ > *E*_sep_, the value of *θ* will be restricted in the dynamical evolution process, while for *E*_0_ < *E*_sep_, there will be no bound. With this definition, the regions with an oscillation phase and a running phase can be well judged. In both regions, *ρ*_0_ oscillates with the oscillation period defined as


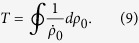


When *E*_0_ = *E*_sep_, the oscillation becomes anharmonic and the period *T* diverges.

**Figure 5:**
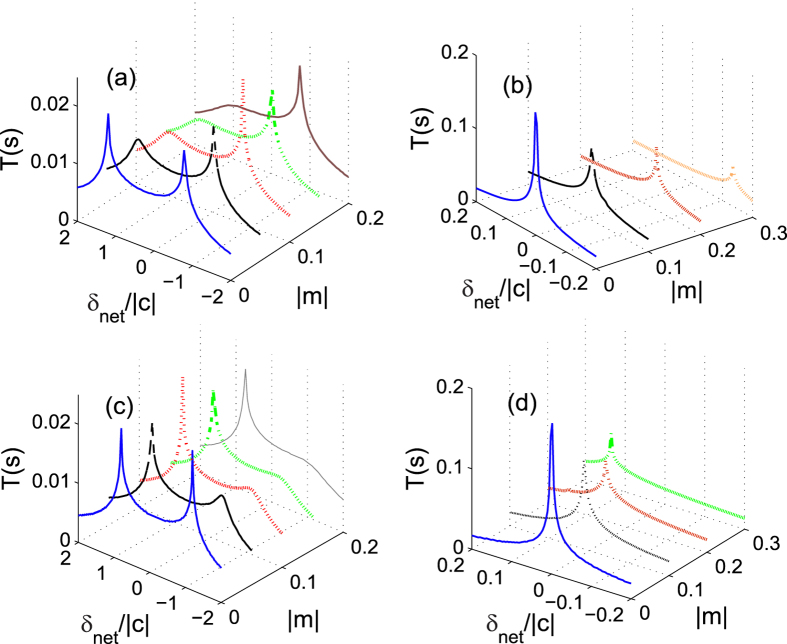
Oscillation period. The dependence of oscillation period *T* on *δ*_net_ and *m* for the (**a**,**b**) ferromagnetic and (**c**,**d**) antiferromagnetic interactions cases. The parameters used here are |*c*| = 52 Hz and the initial conditions are *ρ*_0_ = 0.6 with (**a**,**c**) *θ* = 0 and (**b**,**d**) *θ* = *π*.

For the antiferromagnetic interactions case, *E*_sep_ = *δ*_net_ when *δ*_net_ > 0. We plot the oscillation period *T* as a function of *δ*_net_ and |*m*| in Fig. 5(a–d) for the ferromagnetic and antiferromagnetic interactions cases, respectively with two different initial conditions. For the initial condition with *θ* = 0 in Fig. 5(a,c), there are two peaks of the oscillation period located in the regions *δ*_net_ > 0 and *δ*_net_ < 0, respectively. By contrast, for the initial state with *θ* = *π* in Fig. 5(b,d), we can only find one peak of the oscillation period in the regions *δ*_net_/*c* > 0 and *δ*_net_/*c* < 0 for the ferromagnetic and antiferromagnetic interactions cases, respectively. It can be concluded that the spin oscillation is always harmonic in the other half region of *δ*_net_/*c* without any peak. This observation agrees with the prediction that the spin dynamics in spin-1 spinor condensates substantially depends on the sign of *δ*_net_/*c*^31^.

From Fig. 5 we can also find a remarkably different relationship between the total magnetization *m* and the separatrix in phase space. With the initial condition of *θ* = 0, the position of the separatrix moves slightly when the total magnetization is varied in the positive *δ*_net_/*c* region. In the negative *δ*_net_/*c* region, the separatrix quickly disappears when *m* is away from zero. For the initial condition with *θ* = *π*, the position of the separatrix displays a strong dependence on the magnetization |*m*|. As magnetization |*m*| increases, the position of the separatrix moves to a larger value of *δ*_net_. In fact, the spin dynamics in the negative region for our antiferromagnetic interaction has been realized in a recent experiment^12^, which are similar to those reported with ferromagnetic spinor in magnetic fields where *δ*_net_ > 0^4^,^13^. However, the relationship between the separatrix and *m* for the ferromagnetic system with both positive and negative values of *δ*_net_ has not been experimentally explored yet. We hope that our results for the ferromagnetic interaction case can be realized in future experiments.

## Discussion

In conclusion, we have studied the ground state properties of a spin-1 condensate in a microwave dressing field. Three distinct phases in the MF ground state are demonstrated based on the fractional population of the hyperfine state *m*_*F*_ = 0. For the antiferromagnetic interactions case, there is a phase transition between the BA and AFM phases in the positive *δ*_net_ region. When *m* = 0, the ground state stays in the polar phase for positive *δ*_net_. In the negative *δ*_net_ region the system always stays in the AFM phase. By contrast, for the ferromagnetic interactions case, the phase transition occurs between the BA and AFM phases in the negative *δ*_net_ region. The ground state stays in the BA phase in the positive *δ*_net_ region except for the situation of *m* = 0 and *δ*_net_ ≥ 2|*c*|. The results of the atom number fluctuations show that the *m*_*F*_ = 0 state exhibits a super-Poissonian distribution in the AFM phase for both cases of ferromagnetic and antiferromagnetic interactions. In the BA phase, the *m*_*F*_ = 0 state displays a sub-Poissonian distribution, while in the polar phase, there is no fluctuation.

Moreover, the dynamical properties for different initial conditions are also studied. With the initial condition of *θ* = 0, the position of the separatrix is nearly independence of the total magnetization in the positive *δ*_net_/*c* region. In the negative *δ*_net_/*c* region, the separatrix quickly disappears when *m* is away from zero. For the initial condition of *θ* = *π*, there is no separatrix in the negative *δ*_net_/*c* region. In the positive *δ*_net_/*c* region, the position of the separatrix exhibits a strong dependence on the magnetization |*m*|. Comparing the results for both cases of ferromagnetic and antiferromagnetic interactions, our results convince the prediction that spin mixing dynamics in spin-1 condensate strongly depends on the sign of *δ*_net_/*c*.

## Methods

The static properties for the spin-1 condensate can be studied by numerically solving the eigenquation 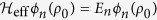, where *n* = 0, 1, 2,... labels the states with increasing eigenenergy. According to the eigenfunction *ϕ*_*n*_(*ρ*_0_), we can obtain the atom number distributions for different stationary states. In the numerical approach, the term 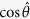 is implemented as 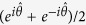, i.e., as a superposition of the left- and right-shift operators in the *ρ*_0_ representation. Then the effective Hamiltonian 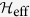 can be expressed as a symmetric tridiagonal matrix and calculated by the numerical diagonalization. Since the spin-exchange collisions couple the states with definite parities, the space of even particle number for spin populations in the hyperfine state *m*_*F*_ = 0 is decoupled from the odd one. Thus, the Hilbert space of the system can be divided into two subspaces. Without loss of generality, in our calculations, we have restricted ourselves to the subspace of even-particle number, in terms of which the atom number is always even in the hyperfine state *m*_*F*_ = 0.

## Additional Information

**How to cite this article**: Huang, Y. *et al.* Phase diagram and spin mixing dynamics in spinor condensates with a microwave dressing field. *Sci. Rep.* **5**, 14464; doi: 10.1038/srep14464 (2015).
